# Response of follicular dendritic cell sarcoma to gemcitabine and docetaxel: report of two cases and literature review

**DOI:** 10.1186/2045-3329-4-6

**Published:** 2014-06-28

**Authors:** Robert M Conry

**Affiliations:** 1 Division of Hematology & Oncology, University of Alabama at Birmingham, 2145 Bonner Way, Birmingham, AL 35243, USA

**Keywords:** Follicular dendritic cell, Sarcoma, Gemcitabine, Docetaxel, Chemotherapy, Metastatic

## Abstract

Follicular dendritic cell sarcoma is a rare malignancy arising from follicular dendritic cells, which form a meshwork within lymphoid follicles. Traditional treatment of metastatic disease with CHOP chemotherapy, the most commonly used regimen for lymphoid malignancies, has met with limited success. I report herein two cases of follicular dendritic cell sarcoma metastatic to the liver treated with the combination of gemcitabine and docetaxel showing unprecedented response in this disease. These observations coupled with recent evidence of a mesenchymal origin of follicular dendritic cells support the premise that this malignancy should be treated similar to other adult soft tissue sarcomas.

## Background

Follicular dendritic cell sarcoma (FDCS) is a rare malignancy arising from follicular dendritic cells which form an arborizing meshwork within lymphoid follicles and are involved in antigen capture, retention and presentation to B cells. Follicular dendritic cells (FDCs) also protect against autoimmunity by expediting removal of potentially self-reactive debris from negatively selected, apoptotic B lymphocytes in germinal centers [1]. The hyaline vascular variant of Castleman’s disease, angiofollicular lymph node hyperplasia, involves a premalignant proliferation of FDCs with dysplasia. Approximately 20% of cases of FDCS are associated with this entity with sequential pathological changes from Castleman’s disease to FDCS documented at individual sites in several reported cases [2,3]. Multicentric Castleman’s disease is usually responsive to CHOP chemotherapy or single agent rituximab despite the fact that FDCs are CD20 negative. The likely mechanism involves depletion of follicular B lymphocytes with secondary reduction of lymphotoxin and other mediator release essential for FDC development. Evidence suggests that Epstein-Barr virus plays a causative role in a rare subset of FDCS affecting the liver and spleen [4].

FDCS affects males and females equally with a median age at diagnosis of 47 years, with a wide range from 14 to 77 years [3]. Approximately 60% of cases arise in lymph nodes, most commonly cervical or mediastinal. Some occur in extranodal sites including lung, liver, spleen and GI tract [5]. FDCSs typically present as a large mass with mean diameter ranging from 7 to 10 centimeters. Systemic symptoms are uncommon. Pathologically, FDCSs are well-circumscribed with spindle to ovoid cells arranged in a fascicular, whorled or storiform pattern, typically with infiltration by scattered small lymphocytes. Ultrastructural examination reveals complex cytoplasmic processes and features resembling fibroblasts [6]. Immunohistochemistry is required to make the diagnosis. FDCSs are usually reactive for FDC markers CD21, CD23, and CD35 [5]. Tumor cells also typically express vimentin, a soft tissue sarcoma marker.

Surgical excision is the treatment of choice for localized FDCS with radiation therapy also used in approximately a third of cases. Local recurrences are reported in 30-40% of cases and metastases also develop in approximately 30% of patients. CHOP chemotherapy has been the most frequently reported systemic therapy with transient, partial responses observed in some patients [7]. Complete responses to CHOP are rare, and the benefits of this regimen may derive primarily from doxorubicin, one of the most broadly active agents against sarcoma in general.

The regimen of gemcitabine and docetaxel is broadly active against soft tissue sarcomas with relatively good tolerability [8]. Gemcitabine 900 mg/m^2^ is administered days 1 and 8 with docetaxel 100 mg/m^2^ administered day 8 of a 21 day cycle with neulasta, G-CSF, given day 9. Response rates are improved using a fixed dose rate infusion of gemcitabine at 10 mg per m^2^ per minute [9], although cytopenias are somewhat more pronounced. Although objective responses occur in 50% of leiomyosarcomas, the overall response rate is approximately 20% in previously treated soft tissue sarcomas in general [8]. A multicenter phase II trial compared gemcitabine with or without docetaxel and the combination produced superior objective response rate, progression-free survival and overall survival [10]. Although RECIST responses occured in only 16% of patients receiving the combination, median progression-free survival was 6.2 months [10]. I report hereafter two cases of FDCS metastatic to the liver with objective response to gemcitabine and docetaxel.

## Case report

Patient 1 was a 51-year-old Caucasian male who underwent wide excision of a FDCS of the duodenal wall with negative margins. This was a spindle cell malignancy with immunohistochemistry positive for CD21, CD23 and vimentin and negative for CD20, CD34, CD35, CD117 (c-kit), DOG 1 and smooth muscle actin. EGFR expression was negative by immunohistochemistry and mutation analysis was wild type. One year after surgery, he was diagnosed with multiple liver metastases confirmed by biopsy and received six cycles of CHOP chemotherapy at another medical center with partial response. However, four months after completing CHOP he had marked worsening of hepatic metastases with numerous lesions affecting all hepatic segments up to 7 cm diameter. Prior to transfer of care to our center, he went untreated for two additional months during which time liver metastases coalesced with the largest becoming 15 cm in diameter, but the lungs remained clear. He had moderate right upper quadrant pain and fullness, but surprisingly no constitutional symptoms such as fatigue, anorexia or weight loss. Due to limited tissue availability, only two genes were sequenced to determine potential clinical trial eligibility, and both BRAF and PI3KCA were wild type as has been reported in FDCS. He received gemcitabine by fixed dose rate infusion and docetaxel since he had already approached his lifetime limit of doxorubicin. After three cycles, he achieved a RECIST 1.1 partial response with the largest hepatic metastasis decreasing from 15 to 9.5 cm diameter, the second largest decreasing from 10.3 to 7.0 cm and symptomatic improvement. After six cycles, the hepatic metastases continued to respond with the largest mass decreasing to 6.4 cm and the second largest to 5.9 cm. Docetaxel was discontinued after six cycles due to lower extremity edema and bilateral pleural effusions, which subsequently resolved. Gemcitabine was continued for a total of 12 cycles with the largest hepatic mass decreasing to 5 cm and the second largest to 4.7 cm. Performance status and quality of life remained excellent throughout treatment, and a RECIST 1.1 partial response is continuing at the time of this report.

Patient 2 was a 36-year-old African-American female who presented with Coombs positive autoimmune hemolytic anemia unresponsive to steroids successfully treated with splenectomy. One year later, she developed generalized abdominal pain with a 9 cm left periaortic, mesenteric mass with open biopsy revealing FDCS with brisk mitoses (25 per 10 hpf). Immunohistochemistry was positive for CD21, CD23, EGFR, vimentin and dim CD35, but negative for CD20, CD34, CD117 (c-kit) and DOG 1. Insufficient tissue was available for gene sequencing. Bone marrow biopsy and aspirate were mildly hypercellular, but otherwise unremarkable with normal karyotype. Her hemoglobin was stably low at 10.5 with normal MCV and a persistently positive direct Coombs test for IgG. Retrospectively, her autoimmune hemolysis requiring splenectomy appears likely related to subclinical FDCS, particularly since FDCs are known to share antigens with erythrocytes, including CD35. Over two months the primary tumor became 30 percent larger and was treated with radiation. Six months after completing radiation therapy, the primary mass had decreased from 8.8 to 6.6 cm with increased central necrosis, but multiple new hepatic metastases had developed up to 3.3 cm with lungs remaining clear. Liver biopsy confirmed metastatic FDCS, and she received fixed dose rate gemcitabine and docetaxel as initial systemic therapy. After six cycles, she achieved a RECIST 1.1 partial response with the largest liver metastasis decreasing from 3.3 cm to 1.8 cm and the second largest decreasing from 2.4 cm to 0.9 cm. After nine cycles of gemcitabine and docetaxel, the largest metastasis further decreased to 1.5 cm and the second largest remained stable at 0.9 cm. The radiated periaortic primary gradually decreased in diameter and developed calcifications. Chemotherapy was discontinued after nine cycles due to plateauing response and fatigue. At the time of this report, she has a continuing RECIST 1.1 partial response two months after discontinuing gemcitabine and docetaxel.

## Discussion

The historic rationale for using CHOP chemotherapy, the most commonly used regimen to treat intermediate grade lymphoma, as systemic therapy for FDCS involved several early observations linking FDCS with B lymphocytes. For example, FDCSs most often arise within lymph node follicles, 20% of cases occur within a background of follicular lymph node hyperplasia, i.e. Castleman’s disease, rare cases are associated with Epstein Barr virus infection, and origin of FDCs from lymphoid precursors was postulated. However, response of Castleman’s disease to CHOP likely involves depletion of B lymphocytes with resultant reduction in paracrine release of FDC growth factors rather than direct cytotoxicity to FDCs. Furthermore, recent work has shown FDC precursors to be of vascular stromal rather than lymphoid origin. FDCs can emerge anywhere in the body during chronic inflammation and participate in the organization of new B cell follicles in non-lymphoid tissues suggesting the existence of a ubiquitous progenitor. Recently discovered FDC precursors are sessile cells originating from vascular stroma found throughout the body in vascular walls and expressing platelet-derived growth factor receptor beta [6]. Thus, FDCs are of mesenchymal origin [11]. FDCSs have complex karyotypes associated with structural abnormalities and loss of multiple chromosomes as seen in the majority of adult sarcomas [12], and the clinical behavior of these tumors is more akin to that of low to intermediate grade soft tissue sarcomas rather than lymphoid malignancies.

This is the first report of objective response of this rare sarcoma to combination chemotherapy with gemcitabine and docetaxel. These agents are generally well tolerated and applicable to patients across a broad range of age and comorbidity. Response of FDCS to this commonly used sarcoma regimen further supports the mesenchymal nature of this malignancy and suggests a role for other agents like pazopanib approved for soft tissue sarcoma therapy.

Epidermal growth factor receptor (EGFR) expression has been reported in normal FDCs, dysplastic FDCs associated with hyaline vascular Castleman’s disease and 16 of 16 cases of FDCS [11]. Yet, no EGFR mutations or gene amplifications were observed. Absence of somatic mutations of KRAS, NRAS, BRAF and PI3KCA in FDCS suggests EGFR inhibition as another potential treatment strategy [11].

## Conclusion

Durable objective response to gemcitabine and docetaxel was observed in two patients with follicular dendritic cell sarcoma metastatic to the liver. This observation supports the notion that FDCS should be treated similar to other adult soft tissue sarcomas rather than lymphoid malignancies. These are to my knowledge the only reported cases of FDCS treated with the combination of gemcitabine and docetaxel. This combination could represent a therapeutic alternative for follicular dendritic cell sarcoma.

### Consent

Written informed consent was obtained from the patients for publication of this Case Report.

## Competing interests

The author declare that they have no competing interests.
